# The case for establishing a Holocaust survivors cohort in Israel

**DOI:** 10.1186/2045-4015-3-22

**Published:** 2014-06-24

**Authors:** Caroline HD Fall, Kalyanaraman Kumaran

**Affiliations:** 1 MRC Lifecourse Epidemiology Unit, University of Southampton, Southampton General Hospital, Tremona Road, Southampton SO16 6YD, UK

## Abstract

In this issue, Keinan-Boker summarises the main studies that have followed up offspring of women exposed to famine during pregnancy and calls for the establishment of a national cohort of Holocaust survivors and their offspring to study inter-generational effects. She suggests that the study would consolidate the fetal origins theory and lead to translational applications to deal with the inter-generational effects of the Holocaust. Barker suggested that alterations in the nutritional supply during critical stages of intra-uterine development permanently alter the structure and metabolism of fetal organs which he termed ‘fetal programming’ (now known as developmental origins of health and disease). The famine studies have played an important role in refining the hypothesis by allowing a ‘quasi-experimental’ setting that would otherwise have been impossible to recreate. The developmental origins hypothesis provides a framework to link genetic, environmental and social factors across the lifecourse and offers a primordial preventive strategy to prevent non-communicable disease. Although the famine studies have provided valuable information, the results from various studies are inconsistent. It is perhaps unsurprising given the problems with collecting and interpreting data from famine studies. Survival bias and information bias are key issues. With mortality rates being high, survivors may differ significantly from non-survivors in factors which influence disease development. Most of the data is at ecological level; a lack of individual-level data and poor records make it difficult to identify those affected and assess the severity of effect. Confounding is also possible due to the varying periods and degrees of food deprivation, physical punishment and mental stress undergone by famine survivors. Nonetheless, there would be value in setting up a cohort of Holocaust survivors and their offspring and Keinan-Boker correctly argues that they deserve special attention. National support is essential as the study may re-open old wounds. The study will need to be appropriately planned and resourced. If properly designed, it may provide further insight into the developmental origins hypothesis and suggest translational applications. It may also influence provision of support to women and children affected by man-made wars and famines that continue to happen across the world.

## 

In her article in this issue (“The mothers have eaten unripe grapes and the children’s teeth are set on edge”) Keinan-Boker reviews the evidence that exposure to undernutrition in utero and during other critical periods in early development has permanent adverse effects on health [1]. She then reviews the findings of studies that have followed up the children of women exposed to famine during pregnancy. Finally, she calls for a national study to be established in Israel to examine the long-term effects of the Holocaust on the survivors and their children. Such a study would fulfil three objectives: 1) to redress a neglected obligation to look after Holocaust survivors by dealing with the inter-generational consequences; 2) to further consolidate the developmental origins theory; and 3) to develop translational applications.

In 1976, Ravelli et al. published a landmark famine study which provided the first strong evidence of long-term effects of undernutrition in fetal life. The authors studied rates of obesity among 300,000 young male army conscripts whose mothers had experienced famine in pregnancy during the Dutch Hunger Winter of 1944-1945 [2]. Famine exposure in early gestation was associated with an increased risk of adult obesity compared to controls. The authors proposed the revolutionary concept that early gestation famine exposure permanently affected the development of hypothalamic centres controlling appetite and food intake, leading to later obesity.

It was not until David Barker put forward his ‘fetal origins hypothesis’ in the 1990’s that these ideas started to create widespread interest and stimulate systematic research. Barker used health visitor records dating back to 1911 from the UK county of Hertfordshire to show that lower birth weight and weight at the age of one year were associated with an increased risk of adult hypertension, type 2 diabetes and death from cardiovascular and lung disease [3,4]. Over several years he formulated the hypothesis that fetal undernutrition, indicated by a low birth weight, permanently changes the structure and metabolism of the body’s tissues, resulting in adult chronic disease [5,6]. He suggested that different organs are affected according to the timing and severity of the insult, reflecting different periods in gestation or early post-natal life when tissues undergo rapid cell division and differentiation. He also proposed that fetal undernutrition permanently alters gene expression; for example leading to tissue-specific insulin resistance as an adaptive mechanism to down-regulate growth in the face of an inadequate nutrient supply. He described these changes as examples of ‘programming’ , a term originally coined to describe permanent effects resulting from transient environmental insults during critical periods of development in experimental animals [7]. The fetal origins hypothesis offered not only a fresh paradigm for the aetiology of adult chronic disease, but also the potential to prevent disease by building better mothers and babies. Barker strongly advocated that nations should invest in the nutrition and health of adolescent girls and pregnant women as an important part of any strategy for combatting the current global epidemics of cardiometabolic disease.

Twenty years later, much research has gone into testing these ideas and much progress has been made. Cohort studies in diverse human populations have replicated the Hertfordshire associations. Other adult health outcomes have been linked to low birth weight, including mental illness, osteoporosis, and chronic kidney disease [8]. Birth weight is now known to be related to functional and ‘human capital’ outcomes in adult life, including height, muscle strength, cognitive ability and educational attainment [9]. Other early life exposures have shown associations with later health including, among others, maternal gestational diabetes (an example of fetal *over*-nutrition) [10], maternal smoking [11] and patterns of infant feeding [12]. Interactions have been demonstrated, between genotype and birth weight, and between birth weight and childhood growth or social circumstances, in relation to later outcomes [13,14]. Research in animals has ‘proved the principle’ that transient manipulation of the mother’s diet in pregnancy can induce diabetes, raised blood pressure, increased adiposity, and other effects in the adult offspring, some of which persist into the next generation [15]. The new science of epigenetics has provided a mechanism by which early-life environmental stimuli could permanently alter gene expression within an individual and inter-generationally [16]. Developmental Origins of Health and Disease (DOHaD) has now replaced Fetal Origins of Adult Disease (FOAD) to describe this field of science, in recognition of the fact that programming effects can occur, and are modified by, events beyond intra-uterine life and infancy. DOHaD has become a framework to link genetic, environmental and social effects on health throughout the lifecourse and inter-generationally.

The DOHaD hypothesis has been controversial from the start, and remains so. While it is generally accepted that early life exposures can have permanent effects, the size of these effects and their importance for human health are still hotly debated. Barker’s recommendation to improve the nutrition and health of girls and young women in order to prevent adult chronic disease in the next generation (some have called this ‘primordial prevention’) has had limited impact at policy level. In most populations, efforts to prevent cardiometabolic disease remain in the realm of ‘secondary prevention’ , principally targeting the overweight and middle aged. This is partly because the evidence for developmental origins of disease is still based mainly on observational data from cohort studies. Several groups of researchers have started to address this by setting up trials of nutritional and other interventions in pregnancy, and following up the children to study long-term health outcomes [17]. However, most of these have follow-up only as far as childhood, and it will take many years before they produce definitive answers. It will take even longer to assess effects in the next generation. Hence the ‘quasi-experimental’ approach of studying the consequences of historic famines and other man-made catastrophes is attractive.

Keinan-Boker summarises the findings from a number of long-term follow-up studies of famine exposure in early life, from around the world. It is important to note others that have recently been published, such as a follow-up of the Nigerian (Biafran) famine of 1967-1970, which showed an increased risk of adult hypertension, impaired glucose tolerance and overweight in those exposed in utero or infancy [18]. Studies on seasonal famines in The Gambia and Bangladesh are also relevant [19]; the Gambia study showed dramatically increased young adult mortality among people born in ‘the hungry season’ , while the Bangladesh study found no such effect. In the Gambia, no differences were found in blood pressure, plasma glucose and lipids, or other cardiovascular risk factors between people born in the ‘hungry’ or ‘harvest’ seasons [20]. Several recent studies have examined famine exposure at older ages (childhood through to young adulthood) and have reported effects on diabetes and cardiovascular disease risk [21-23]. The best known famine studies are the Dutch Hunger Winter studies, which have produced over 80 publications on long-term health effects in the children of women who experienced severe famine during pregnancy [24,25]. The circumstances of this famine were unusual, in that it affected a previously well-nourished population, for a short period of a few months, after which nutrition returned rapidly to normal, making it possible to explore the consequences of exposure during different trimesters of pregnancy. Amazingly, despite the privations, some hospitals managed to continue to record individual-level data, such as birth weight, throughout the famine. Two main research groups have studied the long-term effects, using slightly different exposure definitions and controls. There are several commonalities, and the studies suggest that effects depend on the timing of the famine in gestation. For example, both groups have reported dyslipidaemia and poorer perceived health or quality of life among men and women exposed to famine in early gestation, and an increased risk of type 2 diabetes with any *in utero* exposure, but minimal or no effects on adult mortality or cognitive function. However results in relation to coronary heart disease, hypertension, metabolic syndrome and mental disorders differ between studies. Both have examined effects in the subsequent generation (unexposed children of those exposed to famine in utero) and have shown persisting effects on size at birth [24,25]. While famine studies have been valuable in providing general support for the developmental origins hypothesis, inconsistencies between studies have made firm conclusions about effects of specific periods of exposure impossible, and have limited their translational impact.

There are a number of problems that beset the interpretation of all famine studies. Unsurprisingly, given the context and severity of the effects on populations, routine data collection systems often collapsed, leading to a lack of individual-level data and poor records of who was affected and how much. Even in prolonged, severe famines, some families and individuals probably did better than others; for example the better-off may have been able to afford ‘black market’ food, and rural families may have been able to forage for food. Different individuals may have experienced peak undernutrition at different times. These differences tend to be unrecorded. An important issue for all famine studies is survival bias; mortality rates were high at the time of the famine, and survivors are likely to have differed from non-survivors in ways that could influence their later disease risk. The fact that fitter individuals were more likely to survive may even produce paradoxical findings, with apparent health *advantages* among survivors [25]. A linked issue is selection bias. Amongst the survivors, only a proportion have been followed up in various studies. The possibility of differences between participants and non-participants cannot be ruled out.

The ‘midwife cohort study’ from Guernsey is a good illustration of the difficulties [26]. It was based on babies delivered by one community midwife between 1923 and 1937, and was used to study the effects of undernutrition during German occupation of the island during World War II. Guernsey was occupied from 1940 to 1945, but starvation was at its worst for 9 months in 1944-1945. In order to determine exposure, the midwife records were matched by hand to Guernsey birth registers, to obtain the parish of residence, and to German occupation registers, to identify people who were resident on the island during the occupation (many islanders were evacuated before the occupation). Outcomes were hospital admissions for cardiovascular events, from hospital episode statistics (HES). A total of 113 individuals died before the age of 18 years; a further 457 had no HES record, and it was impossible to determine whether these had left Guernsey (before or after the occupation) or had simply never required hospital admission. Of the 1673 original births, only 225 were resident during the occupation and survived until the HES system began. The study showed an increased risk of cardiovascular events in men and women resident on the island during the occupation, suggesting that exposure to a combination of stress and undernutrition during childhood, adolescence or early adult life was associated with an increased risk of cardiovascular disease. The sample size was too small to examine effects of exposure at different ages. There was no individual-level data available on the severity of the exposure, and it was difficult to exclude the possibility that the families left behind on the island were a more deprived group in the first place than the families who got away.

Many similar issues will threaten the success of the proposed Holocaust survivors study. Firstly, the wide variation in exposures; Holocaust survivors had complex and varying experiences, including acute and chronic mental and physical stress. Many experienced prolonged harassment, the loss of rights, livelihoods and possessions, housing in over-crowded and unsanitary conditions, and severe food deprivation before the extreme terror and even worse conditions of the concentration/death camps. It will be difficult to ascertain and classify these exposure histories, to know when they started and stopped, to disentangle effects of undernutrition and stress, and to make sense of them in a biological way. It is possible that the psychological trauma influenced people’s ability to care for their children for many years afterwards. Even the families that escaped from Europe in time, and who might be considered a control group, are also likely to have experienced stress, either during the escape or on arrival in other countries that were suffering shortages because of the war. A Finnish study has shown an increased risk of type 2 diabetes and cardiovascular mortality among people who were evacuated abroad as children during World War 2 [27].

These difficulties should not put the researchers off, but a Holocaust survivors study will need to be expertly designed, especially with regard to how the complex exposure data are handled. The research questions and analysis plans will need to be tightly formulated at the start, to ensure credibility of the findings and to avoid the risk of data dredging. Positive aspects of setting up a Holocaust survivor cohort in Israel are that if this were a truly national effort, it would be possible to study a large sample of people, to derive unified exposure definitions, to include exposures in childhood and adolescence as well as in utero and in infancy, and because of the individual number system linking Israeli citizens to their health records, to obtain good outcome measures. Importantly, a sample size adequate to study inter-generational effects, in children of people exposed in utero or childhood may be feasible. We would argue that, given the experiences of other famine cohorts, it is worth doing *only* if it is a national effort and adequately planned and resourced. It will also need to have the nation’s will behind it in the sense that the study itself may re-open wounds. It is not easy for the survivors of such terrible events to describe their experiences, or to know that their health is permanently impaired, or (even more) to know that their children and grandchildren may be adversely affected.

We agree, that with all these caveats, a Holocaust survivors cohort would be a worthy enterprise. Keinan-Boker is right that the Holocaust survivors deserve special attention to their health. If designed well, the study could offer a good test of the developmental origins hypothesis. It may be an important step in making the case for better protection of the human rights of women and children caught up in the man-made catastrophes that continue to happen, and provide biological insights that influence the care of women and children in all populations around the world.

## Competing interests

The authors declare that they have no competing interests.

## Biographical statements

Caroline Fall is Professor of International Paediatric Epidemiology at the MRC Lifecourse Epidemiology Unit, University of Southampton, UK. She qualified in Medicine in 1979, and trained in clinical medicine and paediatrics. She joined the MRC Unit in 1989 and was awarded her doctorate for research in the Hertfordshire birth cohort linking lower birth weight with an increased risk of adult diabetes. She now co-ordinates a ‘developmental origins’ research programme in India which includes prospective birth cohort studies and nutritional intervention trials in pregnancy. She was a founder member of the International DOHaD Society (http://www.dohadsoc.org) and its secretary from 2003-2012. Kalyanaraman Kumaran is a public health physician and Senior Lecturer in Epidemiology, based in both the UK (MRC Lifecourse Epidemiology Unit, Southampton) and India (Diabetes Unit, KEM Hospital, Pune), working on projects relating to the developmental origins of health and disease. His interests include the application of evidence-based interventions at population level through a lifecourse approach, reduction of health inequalities, and teaching initiatives.

## Commentary on

Keinan-Boker L. “The mothers have eaten unripe grapes and the children’s teeth are set on edge”: the potential inter-generational effects of the Holocaust on chronic morbidity in Holocaust survivors’ offspring.
